# Flexible Electronics for Monitoring *in vivo* Electrophysiology and Metabolite Signals

**DOI:** 10.3389/fchem.2020.547591

**Published:** 2020-11-19

**Authors:** Hye Kyu Choi, Jin-Ho Lee, Taek Lee, Sang-Nam Lee, Jeong-Woo Choi

**Affiliations:** ^1^Department of Chemical and Biomolecular Engineering, Sogang University, Seoul, South Korea; ^2^School of Biomedical Convergence Engineering, Pusan National University, Yangsan, South Korea; ^3^Department of Chemical Engineering, Kwangwoon University, Seoul, South Korea; ^4^Uniance Gene Inc., Seoul, South Korea

**Keywords:** flexible electronics, biosensor, *in vivo* monitoring, electrophysiological signal, metabolite signal

## Abstract

Numerous efforts have been made to develop efficient biosensors for detecting analytes in the human body. However, biosensors are often developed on rigid materials, which limits their application on skin, organs, and other tissues in the human body where good flexibility is required. Developing flexible materials for biosensors that can be used on soft and irregularly shaped surfaces would significantly expand the clinical application of biosensors. In this review, we will provide a selective overview of recently developed flexible electronic devices and their applications for monitoring *in vivo* metabolite and electrophysiology signals. The article provides guidelines for the development of an *in vivo* signal monitoring system and emphasizes research from various disciplines for the further development of flexible electronics that can be used in more biomedical applications in the future.

## Introduction

A biosensor is a platform for detecting biological components. Applications may range from the medical field to environmental science, and both continuous and intermittent monitoring may be required (Brindha et al., 2018; Khansili et al., 2018; Yang and Gao, 2019). For a decade, researchers have focused on increasing the sensitivity and selectivity of biosensors to improve sensing performance (Lee et al., 2005; Jeong et al., 2018; Park et al., 2018). However, due to the soft and irregularly shaped structure of biological conditions, conventional biosensors developed on rigid materials are not suitable for monitoring signals from surfaces such as skin, organs, and other tissues in the human body (Kim et al., 2019; Salim and Lim, 2019). Therefore, a novel design for biosensors is required to monitor biological components, such as metabolites and electrophysiology signals, more accurately and precisely (Huang and Zhu, 2019; Xu et al., 2019; Yao et al., 2020). A suitable substrate material could conform to the shape of the biological structures without causing adverse effects such as tissue damage and inflammation (Park et al., 2014; Song et al., 2019).

To meet this demand, more research has focused on flexible electronics to expand the application of biosensors in clinical fields, particularly in the healthcare industry (Liu et al., 2018; Chung et al., 2019). Biocompatible and mechanically flexible substrates could be used on delicate, curvilinear interfaces such as the human body (e.g., wearable biosensors) or *in vivo* for monitoring biological components in humans (Boutry et al., 2019; Huang et al., 2019). For example, flexible, synthetic polymers, such as polyethylene terephthalate (PET), polyimide (PI), poly (dimethylsiloxane) (PDMS), and polyethersulfone, and natural polymers including cellulose and silk fibroin have been evaluated as base components for flexible electronic devices for biomedical applications, specifically for implantation (Yao et al., 2019; Park et al., 2020).

**Figure 1:**
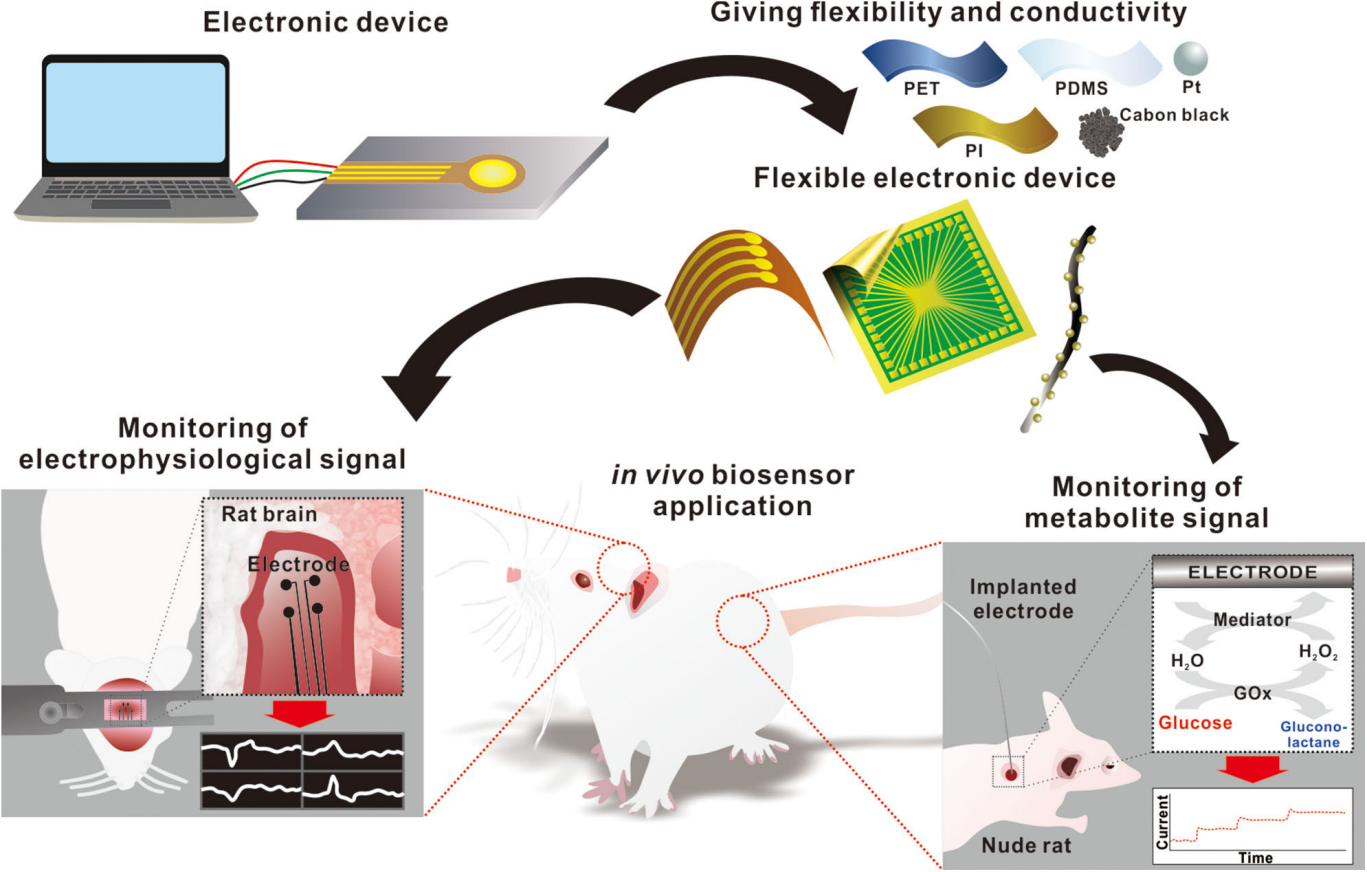
Schematic diagram of flexible electronics for monitoring *in vivo* signals.

Although many reviews discuss flexible electronics for use in wearable biosensors, the recent research on the development of an *in vivo* signal monitoring device warrants a thorough review at this time. This mini-review will focus on some of the current state-of-the-art developments on flexible electronic devices that can be used to monitor *in vivo* electrophysiology and metabolite signals. This article provides a broad overview of the potential for *in vivo* signal monitoring devices for human healthcare, which may be useful for researchers across various disciplines (Figure 1).

## *In vivo* Monitoring of Electrophysiological Signals

Many neuroscientists have studied the structural and functional relationships between the brain and brain potential mapping to understand and treat neurological diseases (Woo et al., 2017; Lake et al., 2018; Murphy et al., 2019). Although, there are many available imaging techniques for brain mapping, such as magnetic resonance imaging (MRI), positron emission tomography (PET), computed tomography (CT), and electroencephalography (EEG), electrical activity in the brain is difficult to monitor using current techniques due to slow response or low resolution (Damborská et al., 2019; Glaab et al., 2019; Hou et al., 2019; Huhn et al., 2019; Klauser et al., 2019). Numerous electronics, such as cortical probes and electrodes, which use penetrating probes or surface electrodes, have been developed with various materials to reliably acquire an accurate electrical signal. However, integrating conventional electrodes into the neural system of living animals is complicated by the differences in the mechanical properties of biological surfaces compared to glass or metal (Ganesana et al., 2019; Ji et al., 2019; Liu et al., 2019; Peng et al., 2019). As a result, the development of electrophysiological electrodes with appropriate mechanical (flexible and soft) properties, biocompatibility, and high electrical/electrochemical performance has become a promising field of research for monitoring and modulating neural interfaces with soft tissues, especially for implantation (Hong and Lieber, 2019; Hossain et al., 2019; Jayant et al., 2019; Das et al., 2020).

In an effort to develop biosensors capable of both *in vitro* and potentially *in vivo* sensing, Pothof et al. fabricated biocompatible stereo-electroencephalography (SEEG) probes (minimal feature size of 5 μm) on a flexible polyimide (PI) foil with platinum electrodes and leads (Pothof et al., 2016). The stability and functionality of the device were tested with saline solution. The PI probe was rolled into a cylindrical shape with a diameter of 0.8 mm, and probes with 32 and 64 electrode sites were implanted 22 mm deep into the posterior parietal cortex of the brain of one monkey (Macaca mulatta), which was trained to perform different motor tasks. Local field potentials (LFP) and multi-unit activity (MUA) were measured 1 h after implantation. Moreover, stable single-unit activity (SUA) was achieved up to 26 d after implantation. Similarly, Kim et al. fabricated a PI-based flexible neural probe for precise site stimulation and recording in the deep brain (Kim et al., 2018). The probe was composed of five electrodes, including ground, stimulation, and three recording electrodes at the inserted tip. Because of its soft and flexible mechanical properties, this probe was expected to be foldable, enabling easy insertion into the deep brain tissue via temporarily used tungsten guide sticks. Due to the flexibility and biocompatibility of the PI and the small size (cross-sectional area) of the electrode, the probe did not cause significant damage to the neural tissue or show evidence of serious immune reactions (high density of macrophage or microglia) for 30 d. The additional ground electrode around the stimulation electrode reduced the leakage power by up to 80% (*in vitro*) and 40% (*in vivo*) and allowed selective site stimulation in the brain. The performance of the probe was tested in animal experiments using rats. The probe showed stable impedance and cyclic voltammogram (CV) response for 30 d under continuous electrical stimulation, and neural spike signals from the subthalamic nucleus (STh) in the 7 mm deep brain were successfully recorded after implantation.

On the other hand, Ji et al. developed a fabrication method of enhanced micro-scale wrinkles on a hyperelastic substrate (Polydimethylsiloxane, PDMS, and Ecoflex) based on oil-pretreating, not only to provide comparable flexibility on the curved cortical surface, but also to improve sensing capability due to the increased surface area (Ji et al., 2019). The wrinkled gold microelectrodes showed promising electrochemical properties with a relatively larger effective surface area (33.5% and 41.6% larger) compared to the flat microelectrodes, and no crack or delamination was observed even after electroplating poly(3,4-ethylene dioxythiophene) polystyrene sulfonate (PEDOT:PSS) and platinum black on the wrinkled microelectrodes. The adhesion and stability of the modified electrodes were tested with 2,500 repetitions of cyclic voltammetry scanning. Neural recording ability was further verified by *in vivo* electrocorticogram (ECoG) signal measurements combined with optogenetics in mice.

Expanding from the brain to other organs, Xue et al. fabricated a PI-based 2D cuff electrode to wrap around the vagus nerve (Xue et al., 2018). Because this nerve provides parasympathetic innervation to human organs, stimulation of the vagus nerve has emerged as a new strategy to treat and modulate cardiac function. Utilizing the bendable property of the contact tips of the device, the electrode sites, which are located on the contact tips, can touch the nerve and selectively record and stimulate the vagus nerve. Among the different kinds of materials (Au, Pt, and Pt-black) tested for electrode and electrochemical measurement (electrochemical impedance spectroscopy (EIS) and CV), the Pt-black exhibited ~30 times larger charge delivery capacity (CDC). The *in vitro* measurements of EIS and CV for Au, Pt, and Pt-black, were 405 kΩ, 41 kΩ, and 10.5 kΩ @1 kHz and 0.81 mC/cm^2^, 4.26 mC/cm^2^, and 25.5 mC/cm^2^, respectively (*n* = 3). During the cell viability test over 24 h, no obvious cell damage was observed. For the *in vivo* experiment, the device was implanted into the right-sided vagus nerve of rats. A biphasic current was used to stimulate the vagus nerve with a frequency of 10 Hz, pulse duration of 300 μs, and varying current stimulus. The result showed that the successful stimulation of the vagus nerve reduced the heartbeat rate by up to 36%, and the heartbeat resumed a regular frequency when stimulation was removed. As these studies show, recent advances in flexible electronics have expanded the *in vivo* application for monitoring electrophysiological signals using devices with suitable mechanical and biocompatible properties that do not cause significant damage to the tissues of interest.

## *In vivo* Monitoring of Metabolite Signals

Glucose is one of the essential metabolites in the human body which functions as fuel for cells (Pellerin and Magistretti, 1994; Simpson et al., 2007; Lee et al., 2012; López-Gambero et al., 2019), and abnormal glucose levels can lead to a range of adverse conditions and diseases including diabetes (Santiago et al., 2007; Chen et al., 2018). Therefore, continuous blood glucose monitoring devices are widely utilized in the diagnosis and treatment of diabetes mellitus to provide information about blood glucose levels (Klonoff, 2005; Bruen et al., 2017; Tripathy and Kim, 2018). However, biofouling, inflammation, fibrosis, and extracellular release of lysosomal contents are crucial barriers for the design of reliable, implantable glucose biosensors. For example, electrochemical glucose biosensors are known to lose their sensitivity as soon as they are implanted. The immobilized enzyme (glucose oxidase, GOD) activity on the surface of the electrode is a fundamental limiting factor for *in vivo* conditions due to the accumulation of H_2_O_2_ in the blood (Pickup et al., 1989). In addition, biofouling such as the eventual adhesion and spreading of macrophages on the sensor surface significantly affects the sensitivity of implanted sensors (Yu et al., 2008). To improve the stability and biocompatibility of implanted glucose sensors, Burugapalli et al. fabricated a biomimetic membrane of PU and gelatin (GE) on the electrode using the electrospinning method to act as a mass transport limiting membrane (Burugapalli et al., 2018). Fibro-porous structured membranes with optimized fiber diameters, pore sizes, and permeability were generated using three different polymer solution concentrations (wt %) (8PU, 12PU, and 6PU10GE). The developed electrode with fibro-porous structured membranes was implanted in rats for an *in vivo* functional efficacy test. While the fibro-porous PU-GE structure with an average pore size of approximately 1.5 mm allowed host cell infiltration, PU structure with a similar pore size (~1 mm) was not permeable to host cells at all. Since the fibro-porous PU membrane successfully acted as a mass transport-limiting membrane, 8PU membranes showed the highest sensitivity for both *in vitro* and *in vivo* conditions. However, the biomimetic PU-GE structure most effectively prevented fibrous capsule formation on the sensor surface, producing the slowest decrement of sensing ability in *in vivo* conditions. Though the advantage of the PU-GE coating was diminished after 9 weeks due to the resorption of GE and replacement with collagen, the results demonstrated that the formation of the mass-transport limiting membrane such as a fibro-porous structure could improve the lifespan of implanted *in vivo* glucose biosensors.

Another approach for increasing the lifespan of *in vivo* glucose biosensors includes employing nanomaterials to maintain enzyme activity. Fang et al. utilized Cu nanoflowers to construct a minimally invasive glucose microelectrode (Fang et al., 2018). Electrodeposited flower-shaped Cu nanostructures on the Pt microelectrode provided a large surface area and a high number of active sites to promote electrocatalysis of glucose on the surface of the electrode. The subsequent Nafion layer improved the anti-interference performance of the sensor devices. Glucose oxidase was immobilized using covalent bonding with glutaraldehyde and bovine serum albumin (BSA). In addition, the biocompatible polyurethane (PU) layer was used as an outer membrane to improve the stability and biocompatibility of the implanted glucose sensors. The outer membrane worked as a limiting diffusion layer to reduce enzyme leaching and also limit the diffusion of glucose relative to oxygen. As a result, *in vitro* electrochemical performance with good sensitivity and a large linear response range was obtained by CV and chronoamperometry (CA). Furthermore, a real-time response to the variation of blood glucose concentration was successfully observed in the *in vivo* implantable experiments using anesthetized rats.

Similarly, Pu et al. used a 3D nanostructure consisting of graphene and platinum nanoparticles on a cylindrical, flexible, substrate-based electrode (polyetheretherketone, PEEK, diameter: 1 mm, length of the fabricated sensor: 5 mm, stiffness: 62.5 kN m^−1^) to enhance the sensitivity of an *in vivo* glucose sensor (Pu et al., 2018). The cylindrical PEEK substrate was pre-modified by (3-aminopropyl) trimethoxysilane (ATPMS) and (3-mercaptopropyl) trimethoxysilane (MPTMS) to achieve a hydrophilic and metal adhesive surface to construct an electrode. After rotated inkjet printing and a synchronous heating technique, microstructures were directly developed on a curved surface, providing a larger active surface compared to the traditional pin-type implantable glucose monitoring system. The *in vitro* experimental results demonstrated a sensing ability ranging from 0 to 570 mg/L of glucose, which aligns with the range of physiological glucose levels. After *in vitro* characterization, an *in vivo* experiment was conducted in which the sensor was implanted into the subcutaneous tissue of a rat. The results demonstrated the ability of the device to monitor glucose continuously in subcutaneous tissue.

In a different study, Zhang et al. developed a self-powered, implantable, skin-like glucose sensor for real-time detection of *in vivo* blood glucose levels (Zhang et al., 2018). Based on the piezo-enzymatic-reaction coupling effect of a GOx@ZnO nanowire array, the device converted the mechanical deformation into a piezoelectric impulse, which acted as both the biosensing signal and electrical power. The skin-like device was implanted in a mouse, and the blood glucose concentration was successfully measured in real time. Chen et al. also presented a skin-like electronic device for non-invasive, *in situ*, and highly accurate intravascular blood glucose monitoring (Chen et al., 2017). The ultrathin (~3 mm), nanostructured glucose sensor with high sensitivity (130.4 mA/mM) was developed by integrating multiple layers, including poly(methyl methacrylate) (PMMA), PI, a nanostructured gold thin film, a transducer layer (PB), and a transfer/glucose oxidase (GOx). Additionally, by incorporating electrochemical twin channels (ETC) and reverse iontophoresis, high-density hyaluronic acid (HA) was inserted into the interstitial fluid (ISF) and raised the ISF osmotic pressure to promote intravascular blood glucose transportation from the vessel. The change in glucose concentration in the ISF resulted in reverse iontophoresis at a low-current level to drive intravascular blood glucose to the skin surface. Then, *in vivo* human clinical trials were conducted with hourly measurements over a 1-day period, and the results showed a good correlation (>0.9) with both ISF and blood glucose levels.

Similarly, Gao et al. used a flexible printed circuit board on a mechanically flexible polyethylene terephthalate (PET) substrate to monitor metabolites and achieved *in situ* monitoring of multiple analytes from sweat, including glucose, lactate, K^+^, and Na^+^ (Gao et al., 2016). Although this experiment was performed on the skin instead of in *in vivo* conditions, the results indicate the successful monitoring of sweat analytes in real time, providing an increased ability to understand and diagnose health conditions. For example, *ex-situ* measurements of Na^+^ levels in sweat substantially increased when the subjects had lost a large amount of water (~2.5% of body weight), which indicates that Na^+^ levels in sweat are a potential biomarker for monitoring dehydration. Although body fluids including sweat, saliva, and tears are considered potential candidates for glucose monitoring, the density of glucose in those fluids is quite low compared to blood (only ~1-10%). In addition, the accuracy of fluid glucose measurements is significantly affected by several factors, such as water evaporation and other internal components. Considering the complex physiology of the human body, the developed device will have to overcome several limitations before it can be applied to an *in vivo* system. Furthermore, multiplexed sensing of other metabolite biomarkers would be a key advancement in healthcare monitoring in *in vivo* conditions as well (Chung et al., 2019; Zhang et al., 2020). Glutamate, which is one of the major excitatory neurotransmitters in the central nervous system, has been investigated through flexible electronics in an *in vivo* condition as well (Cao et al., 2012; Nguyen et al., 2019). Additionally, lactate, which is the one of energy metabolites, can also be a suitable candidate for metabolite biomarkers in *in vivo* monitoring (Weltin et al., 2014a,b).

## Concluding Remarks and Future Prospects

Due to their unique properties, flexible electronic devices have gained interest as a promising platform for non-invasive, real-time, and continuous monitoring systems in the healthcare field. In this review, we have summarized some of the most advanced developments in flexible electronic devices to monitor *in vivo* electrophysiology signals and metabolite (e.g., glucose) signals (Table 1). The major advantage of *in vivo* metabolite signal monitoring, compared to *in vitro* monitoring methods, is the continuous and real-time monitoring of the abnormal level of a metabolite which could be an indicator of impending disease. For example, as the abnormal level of glucose can lead to diseases including diabetes, the rapid, sensitive, and continuous monitoring of glucose is critical. Although current *in vitro* glucose monitoring systems are highly sensitive and selective, continuous and real-time monitoring is not currently possible due to fundamental obstacles such as the requirement of sufficient amounts of continuous biological fluids (i.e., blood, or sweat) for reliable monitoring. Furthermore, continuous and real-time monitoring could lead to a personalized care system based on individualized reports such as pharmacokinetic drug effect.

**Table 1:** Flexible electronic devices for *the* monitoring of electrophysiological and metabolite signals.

**Monitoring signal**	**Type of devices**	**Components of devices**	**Tested in**	**Target**	**References**
Electrophysiological signal	Penetrating probe	Polyimide, Ti/Au, Ti/Pt	*In vivo*	Neural spikes from rat brain	Kim et al., 2018
		Polyimide, cytop	*In vivo* and *in vitro*	Single-unit and multi-unit activities, and local field potentials from monkey brains	Pothof et al., 2016
	Surface electrode	PEDOT:PSS, Pt-black	*In vivo*	Electrocortigram signals from somatomotor and somatosensory cortexes	Ji et al., 2019
		Polyimide, Pt-black	*In vivo*	Vagus nerve signal from rats	Xue et al., 2018
Metabolite signal	Penetrating probe	Polyurethane, gelatin	*In vivo*	Blood and interstitial glucose	Burugapalli et al., 2018
		Polyurethane, Cu nanoflower	*In vivo*	Blood glucose	Fang et al., 2018
	Surface electrode	PEEK, graphene, and platinum nanoparticles	*In vivo*	Blood glucose	Pu et al., 2018
		ZnO nanowire, kapton	*In vivo*	Blood glucose	Zhang et al., 2018
		PMMA, polyimide, prussian blue	*In vivo*	Intravascular blood glucose	Chen et al., 2017
		PET	*In vivo*	Glucose, lactate, K^+^, NA^+^	Gao et al., 2016

Although various synthetic and natural materials are being utilized to design better flexible biosensors, some major limitations remain for *in vivo* application. For example, to be effective in clinical applications, overall devices should be designed for minimally invasive implantation and the base material must be flexible and soft to avoid tissue-substrate mechanical mismatch. The mechanical properties of conventional electrodes are not suitable for biological tissues due to their high modulus and stiffness, which can cause damage to the tissues. In addition, the electronic system is supposed to be bendable and flexible to be utilized in an *in vivo* condition. Materials based on flexible and bending properties with stiffness could sufficiently allow us to monitor the micro-motion related to physiological processes including heartbeat and respiration, which is not critical or required for skin applications.

The flexible *in vivo* signal monitoring device should be completely non-toxic, while maintaining its sensitivity, selectivity, and reproducibility compared to conventional biosensors developed on rigid materials. To monitor the metabolites in an *in vivo* condition, the flexible electronics have to be implanted in the body. Therefore, if the flexible electronic system is composed of toxic or non-biocompatible components, it could lead to harmful side effects such as tissue necrosis. For example, due to the complex matrix and harsh conditions inside the body, the degradation of system components can occur. The degradation of the system could release toxic chemicals and cause unexpected side effects. Thus, for the development of flexible electronics for *in vivo* applications, material selection should be made cautiously to avoid any side effects. However, as the degradation property is not crucial for flexible electronics on the skin, there are more possibilities in the choice of the material component. In addition, challenges in the fabrication of flexible *in vivo* signal monitoring devices for continuously monitoring body fluids do not only include the sensing components, but also integrate different technical components, such as fluid delivery, power source, etc. For example, energy consumption will be a limitation in continuous, long-term monitoring. Although many aspects of flexible electronic devices for *in vivo* metabolites monitoring require improvement, these advancements could have a significant impact on biological, biochemical, and medical applications, especially for continuous monitoring in healthcare.

## Author Contributions

The manuscript was written through the contributions of all authors. All authors have read and agreed to the published version of the manuscript.

## Conflict of Interest

S-NL was employed by the company; Uniance Gene Inc. The remaining authors declare that the research was conducted in the absence of any commercial or financial relationships that could be construed as a potential conflict of interest.
